# Adherences to oral nutritional supplementation among hospital outpatients: An online cross-sectional survey in Japan

**DOI:** 10.1371/journal.pone.0222972

**Published:** 2019-09-26

**Authors:** Naoki Hashizume, Yoshiaki Tanaka, Suguru Fukahori, Shinji Ishii, Nobuyuki Saikusa, Yoshinori Koga, Naruki Higashidate, Daisuke Masui, Saki Sakamoto, Minoru Yagi

**Affiliations:** 1 Department of Pediatric Surgery, Kurume University School of Medicine, Kurume, Fukuoka Japan; 2 Division of Medical Safety Management, Kurume University Hospital, Kurume, Fukuoka, Japan; University of Mississippi Medical Center, UNITED STATES

## Abstract

Oral nutritional supplements (ONS) are multi-nutrient products used to increase the energy and nutrient intakes of patients. The aim of this study was to examine whether or not the adherence of patients varies according to their receiving prescription or over-the-counter ONS. Data were obtained from an online cross-sectional survey conducted with patients in Japan. A total of 107 patients who matched the inclusion criteria for the prescription ONS group and 148 who matched the criteria for the over-the-counter ONS group were further analyzed. In the prescription and over-the-counter ONS groups, the main medical reason for ONS consumption were “malnutrition” (48 patients [44.9%] vs. 63 patients [42.6%] p = 0.798], “frailty” (29 patients [27.1%] vs. 36 patients [24.3%] p = 0.663) and “aging” (25 patients [23.4%] vs. 30 patients [20.3%] p = 0.644). The proportion of “No particular disease” for prescription ONS consumption was significantly lower than that for over-the-counter ONS (6 patients [5.6%] vs. 24 patients [16.2%] p = 0.001). The body mass index of the prescription ONS group was significantly higher than that of the over-the-counter ONS group (21.1±4.38 kg/m^2^ vs. 19.9±3.75 kg/m^2^, p = 0.0161). In the prescription ONS group, all patients were given medical advice by doctors or registered dietitians. In contrast, in the over-the-counter ONS group, only 46 patients (31.1%) were given advice by doctors or registered dietitians (p<0.001). In the prescription ONS group, ONS was taken significantly more times and for a longer duration than in the over-the-counter ONS group (p<0.0001). However, among patients given advice by doctors or registered dietitians, there were no significant differences between the groups. Greater support by the medical team is still needed in order to maximize adherence to supplementation, especially concerning the calories, timing and period, so that benefits can be achieved and sustained.

## Introduction

Oral nutritional supplements (ONS) are multi-nutrient products (ready-made liquid, pudding or powder to be mixed with fluids) used to increase the energy and nutrient intakes of patients, especially those with malnutrition and at nutritional risk [1]. The European Society for Clinical Nutrition and Metabolism (ESPEN) introduced the concept of ONS to the ESPEN guidelines on enteral nutrition [2]. ONS are defined as supplementary oral products consumed along with the normal diet for special medical purposes.

In meta-analyses, ONS have been shown to be clinically effective in some patient groups [3–7], such as malnourished geriatric patients [3,4], whereas a Cochrane review on disease-related malnutrition found no major differences in morbidity or mortality between patients receiving dietary advice and those prescription ONS [8]. The ESPEN guidelines strongly recommend that malnourished polymorbid medical inpatients or those at high risk of malnutrition who can safely reach their nutritional requirements orally be considered for ONS high in energy and protein in order to improve their nutritional status and quality of life. It further recommends that nutrient-specific ONS be administered to malnourished polymorbid medical inpatients or those at high risk of malnutrition when they may maintain muscle mass, reduce mortality or improve their quality of life with such a prescription, and that ONS be considered for polymorbid medical inpatients who are malnourished or at high risk of malnutrition and can safely reach their nutritional requirements orally as a cost-effective intervention method for improving outcomes. A variety of benefits have been found for ONS use, including reduced length of stay [9,10], inpatient episode cost [10], complication rates, [11,12] depressive symptoms [13], and readmission rates [14,15], and improved lean body mass recovery [16]. However, the use of ONS has also been questioned due to low adherence [17,18] and a lack of beneficial results for some patient groups [19]. The effectiveness of nutrition therapy using ONS varies due to unstable patient adherence to the prescription, but a higher adherence to ONS has been associated with a higher energy intake [20,21] and an increase in body weight [21].

ONS often contain macronutrients as energy and protein and micronutrients as vitamins and minerals at varying concentrations. ONS that are registered as pharmaceuticals are only available by prescription, ideally following advice from a doctor. Therefore, individual dietetic assessments take into account a patient’s nutritional requirements in order to ensure a tailored prescription. However, some ONS that are registered as foodstuffs are available as over-the-counter purchases in supermarkets or pharmacies without doctors or registered dietitians in Japan. No previous studies have compared the outcomes of two types of ONS “prescription versus over-the-counter ONS”.

The aims of this study were the examination of difference between outpatients consumed prescription ONS and those consumed over-the-counter ONS and adherence to prescription ONS prescribed by a doctor and to over-the-counter ONS purchased by themselves.

## Materials and methods

### Study design

Data were obtained from an online cross-sectional survey conducted with patients in Japan. The survey was hosted by the market research company EPOCA Marketing Co., Ltd., which recruited samples from 2.2 million people registered with the company intended to be representative of the Japan population. Prescription ONS were registered in Japan as follows; Elental^®^ (EA Pharma Co., Ltd, Japan), Elental P^®^ (EA Pharma Co., Ltd, Japan), Ensure Liquid^®^ (Abbott Japan Co., Ltd., Japan), Ensure H^®^ (Abbott Japan Co., Ltd.), Enevo^®^ (Abbott Japan Co., Ltd.), Twinline-NF^®^ (Otsuka Pharmaceutical Co., Ltd., Japan), Racol-NF^®^ (Otsuka Pharmaceutical Co., Ltd., Japan). Over-the-counter ONS are registered as foodstuffs ONS in Japan.

The age distribution rate for prescription ONS was examined using the 2^nd^ National Database of Health Insurance Claims and Specific Health Checkups of Japan (NDB) built by the Ministry of Health, Labor and Welfare of Japan [22]. Given these percentages, questionnaires were collected from the web until the number of patients consuming prescription ONS (including those who also consumed over-the-counter ONS) and those of patients consuming only over-the-counter ONS reached 150 each. The NDB consists mainly of health insurance claims, including basic patient information, such as sex and age, plus items such as the number of insurance points, the name of the illness or injury, medical practice information and drug administration and prescription information.

Patients who consumed prescription ONS combined with over-the-counter ONS and nasogastric tube or gastrostomy were then excluded. Ultimately, the patients who consumed prescription ONS and those who consumed over-the-counter ONS were defined as the prescription ONS group and over-the-counter ONS group, respectively.

### Ethical approval and studies and informed consent

Respondents had to fill out their name in the questionnaire or had to be identifiable in order to be included in this study and prevent data duplication. Respondents’ confidentiality was guaranteed, and privacy policy statements were included in the introduction section of the questionnaire. The study protocol was approved by the Kurume University Ethics Committee (No. 18098).

### Data collection

The questionnaire was started when respondents answered "Yes" to the two following questions: “Have you (or a person in your care) visited a hospital for some illness within the past year?” and “Do you (or a person in your care) currently consume ONS as a hospital outpatient?” The questionnaire then inquired as to whether the respondent was a patient themselves or a caregiver. If the respondent was a caregiver, it asked what their relationship was with the patient. Finally, the survey instrument contained questions about a variety of patient demographic characteristics, such as the age, gender, body mass index (BMI), region, employment and household income (JPY per month). The second part consisted of 18 questions regarding the management and adherence to taking ONS (Table 1).

**Table 1:** Survey questions (excluding demographic questions).

	Survey questions
Q1	What is the medical reason for taking the ONS?
Q2	What is the ONS type and brand?
Q3	How much does the ONS cost (JPY per month)?
Q4	Who recommended that the ONS be taken?
Q5	Were you given advice by doctors or registered dietitians?
Q6	How often were you advised to take ONS? (e.g. daily, once a week, etc.)
Q7	How many calories of ONS were you advised to consume daily?
Q8	How long were you advised to take ONS?
Q9	How often do you actually take ONS? (e.g. daily, once a week, etc.)
Q10	How many calories of ONS do you consume daily?
Q11	How long have you been taking ONS?
Q12	Do you follow the medical advice (timing, amount, duration)?
Q13	If not, why do you not follow the medical advice?
Q14	Did you provide support to make it possible to continue taking ONS?
Q15	Are you satisfied with your ONS (overall, nutrition, ease of consumption, taste, price, ease of prescription/purchase)?
Q16	Are you aware of other ONS?
Q17	If Q16”yes”, have you ever been recommended to take another ONS?
Q18	If Q16”yes”, why do you take the prescription (or over-the-counter) ONS (free comment)?

If respondents answered "Yes" to Q5 “Were you given advice by doctors or registered dietitians?”, the respondents went on to answer Q6-Q8: “How often were you advised to take ONS? (e.g. daily, once a week, etc.)”, “How many calories of ONS were you advised to consume daily?” and “How long were you advised to take ONS?”

If respondents did not answer “Not determined” or “Unknown” for Q6-Q8, the respondents were then asked Q12 “Do you follow the medical advice (number of times, amount, duration)?” Q12 was then analyzed for each of the different dimensions related to following advice concerning the number of times, amount and duration using a Likert-type 4-point scale: not at all well, slightly well, well and very well. Responses to Q15 “Are you satisfied with your ONS (overall, nutrition, ease of consumption, taste, price, ease of prescription/purchase)?” were analyzed for each of the different dimensions related to satisfaction regarding overall satisfaction, nutrition, ease of drinking, taste, price, ease of obtaining using a Likert-type 7-point scale: very dissatisfied, dissatisfied, slightly dissatisfied, neither satisfied nor dissatisfied, slightly satisfied, satisfied, very satisfied and unknown.

Comparison analyses of the surveys were performed between the prescription and over-the-counter ONS groups. Responses to Q9 and Q11 were also compared between the two groups among patients who were given advice by doctors or registered dietitians. Response to Q10 were compared between the two groups after excluding patients who did not know the amount or type of ONS they consumed.

### Statistical analyses

Continuous data were presented as mean ± standard deviation, and categorical data were expressed as the number (%). Group differences were tested using the chi-squared test and the Mann-Whitney U test. All of the statistical analyses were performed using the JMP software package (SAS, Cary, NC, USA), and p values of < 0.05 were considered statistically significant.

## Results

### Demographic characteristics

**Fig 1:**
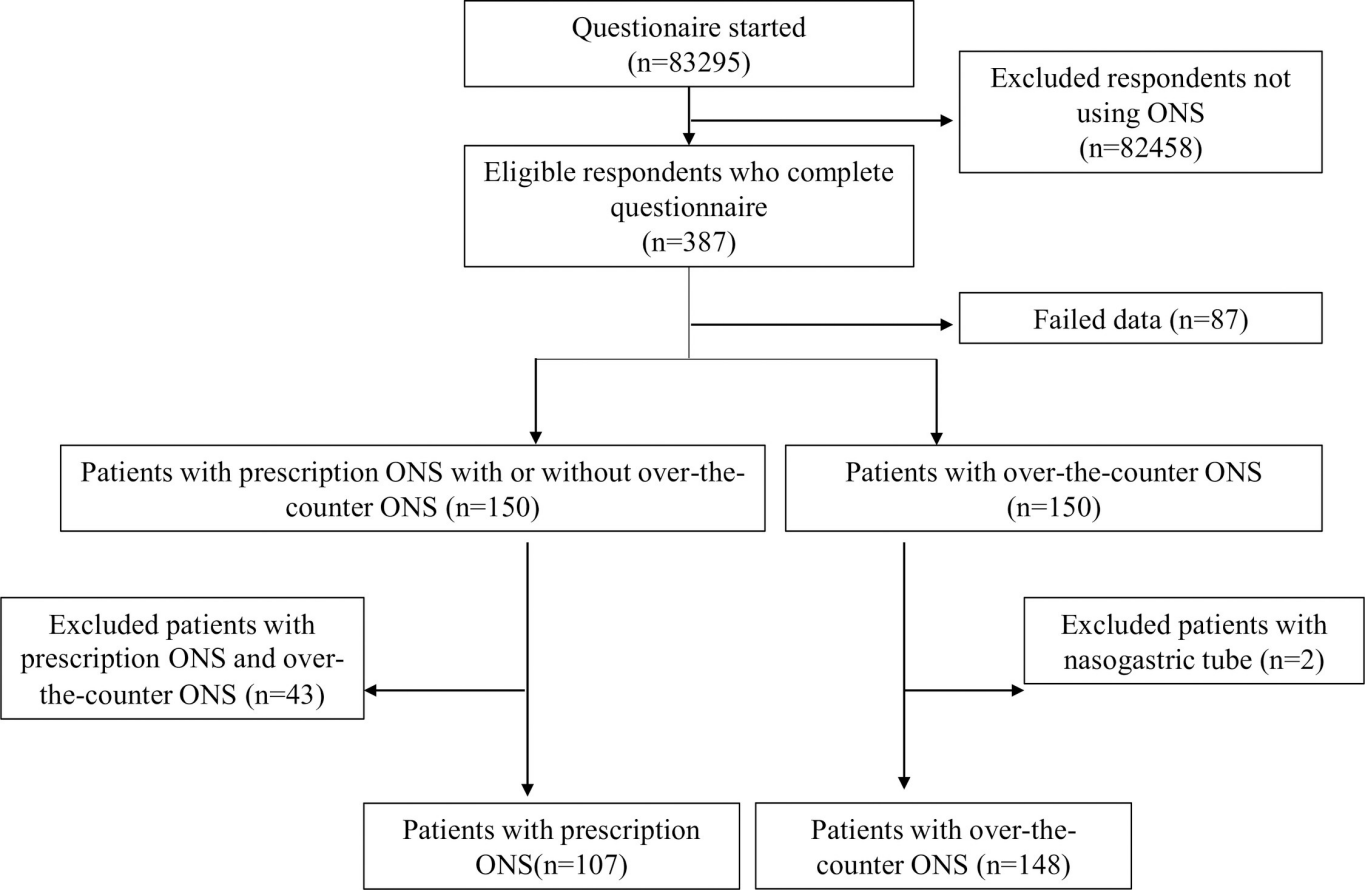
Flow chart of participants in the overall survey.

A total of 14.0% of patients prescribed ONS were ≤50 years of age, 22.0% were 51–74 years of age, and 64.0% were ≥75 years of age in the NDB data. Given these percentages, patients were collected. Responses were collected from August 31 to September 18, 2018 until the number of patients consuming prescription ONS (including those who also consumed over-the-counter ONS) and those of patients consuming only over-the-counter ONS reached 150 each. Fig 1 shows the respondents flow of this study, with 83925 potential respondents sent a questionnaire. As 82456 respondents did not consume ONS, those were excluded. Three hundred and eighty-seven of these patients had consumed ONS within the past year. Eighty-seven patients had insufficient data. Patients who consume prescription ONS and patients consuming only over-the-counter ONS reached 150 each. Of the patients who consumed prescription ONS, 43 patients who combined with over-the-counter ONS were excluded. Of the patients who consumed over-the-counter ONS, 2 patients who used a nasogastric tube were excluded. Ultimately, 107 patients who matched the inclusion criteria for the prescription ONS group and 148 who matched the criteria for the over-the-counter ONS group were further analyzed (Fig 1).

Table 2 reveals the respondents of the questionnaire. In the prescription ONS group, 36 respondents (33.6%) were patients themselves, and 71 (66.4%) were caregivers, and in the over-the-counter ONS group, 53 respondents (35.8%) were patients themselves, and 95 (64.2%) were caregivers. Caregivers were almost always the parent of the patient (Table 2). There was no marked difference in respondents between the prescription and over-the-counter ONS groups.

**Table 2:** Respondent characteristics.

	Prescription ONS	Over-the-counter ONS	*p value*
Respondents, n (%) ^a^			
Patients	36 (33.6)	53 (35.8)	0.790
Caregivers	71 (66.4)	95 (64.2)	
Family position of patients for caregiver, n (%)^b^			
Mother or father	67 (94.4)	87(91.6)	0.656
Grandmother or grandfather	0 (0.0)	1 (1.1)	
Partner	4 (5.6)	4 (4.2)	
Son or daughter	0 (0.0)	0 (0.0)	
Brother or sister	0 (0.0)	1 (1.1)	
Uncle or aunt	0 (0.0)	1 (1.1)	

^a^Prescription ONS (n = 107), over-the-counter ONS (n = 148)

^b^Prescription ONS (n = 71), over-the-counter ONS (n = 95)

Table 3 reveals the demographic characteristics of the patients analyzed. There were no significant differences in the age, sex. The BMI of the prescription ONS group was significantly higher than that of the over-the-counter ONS group (21.1±4.38 kg/m^2^ vs. 19.9±3.75 kg/m^2^, *p* = 0.016). Thirty-five patients in the prescription ONS group (32.7%) and 61 in the over-the-counter ONS group (41.2%) had a BMI <18.5 kg/m^2^.

**Table 3:** Patients’ characteristics.

	Prescription ONS (n = 107)	Over-the-counter ONS (n = 148)	p value
Age,n (%)			
<65	18 (16.8)	32 (21.6)	0.666
65–75	19 (17.8)	21 (14.2)	
>75	70 (65.4)	95 (64.2)	
Sex,n (%)			
Male	54 (50.5)	59 (39.9)	0.098
Female	53 (49.5)	89 (60.1)	
Body mass index,mean±SD (kg/m2)	21.1±4.38	19.9±3.75	0.016
≤18.5 kg/m2,n (%)	35 (32.7)	61 (41.2)	0.120
18.5–25 kg/m2,n (%)	57 (53.3)	72 (48.6)	
25–30 kg/m2,n (%)	9 (8.4)	13 (8.8)	
30–35 kg/m2,n (%)	5 (4.7)	2 (1.4)	
35 kg/m2,n (%)	1 (0.9)	0 (0.0)	

Table 4 reveals region, employment and household income between the groups. There were no significant differences in employment and household income. However, there were significant differences in the region between the two groups. (p = 0.042)

**Table 4:** Region,employment and household income of patients.

	Prescription ONS (n = 107)	Over-the-counter ONS (n = 148)	p value
Region,n (%)			
Hokkaido	3 (2.8)	6 (4.1)	0.042
Tohoku	3 (2.8)	8 (5.4)	
Kanto	42 (39.3)	62 (41.9)	
Chubu	23 (21.5)	24 (16.2)	
Kansai	26 (24.3)	21 (14.2)	
Chugoku	0 (0)	10 (6.8)	
Shikoku	3 (2.8)	2 (1.4)	
Kyushu	7 (6.5)	15 (10.1)	
Employment,n (%)			
full-time	38 (35.5)	47 (31.8)	0.658
self-employment	14 (13.1)	14 (9.5)	
part-time	13 (12.1)	20 (13.5)	
house keeper	9 (8.4)	22 (14.9)	
umemployment	12 (11.2)	19 (12.8)	
retirement	19 (17.8)	25 (16.9)	
other	2 (1.9)	1 (0.7)	
Household income (JPY),n (%)			
<3,000,000	29 (27.1)	39 (26.4)	0.590
3,000,000–5,000,000	26 (24.3)	47 (31.8)	
5,000,000–7,000,000	19 (17.8)	20 (13.5)	
7,000,000–10,000,000	16 (15.0)	21 (14.2)	
10,000,000–15,000,000	9 (8.4)	12 (8.1)	
>15,000,000	8 (7.5)	9 (6.1)	

### Medical reasons for taking ONS (review Q1)

In the prescription and over-the-counter ONS groups, the main medical reason for ONS consumption were “malnutrition” (48 patients [44.9%] vs. 63 patients [42.6%] p = 0.798], “frailty” (29 patients [27.1%] vs. 36 patients [24.3%] p = 0.663) and “aging” (25 patients [23.4%] vs. 30 patients [20.3%] p = 0.644). The statistically significant reasons for ONS consumption were “liver disease” (only in the prescription ONS group, 9 patients [8.4%] p <0.0001), “inflammatory bowel disease” (only in the prescription ONS group, 5 patients [4.7%] p = 0.012). The proportion of “No particular disease” for prescription ONS consumption was significantly lower than that for over-the-counter ONS (6 patients [5.6%] vs. 24 patients [16.2%] p = 0.001) (Table 5).

**Table 5:** Medical reason for taking ONS (Q1).

	Prescription ONS (n = 107)	Over-the-counter ONS (n = 148)	p value
Medical reason for taking ONS (Q1), n (%)			
malnutrition	48 (44.9)	63 (42.6)	0.798
frail	29 (27.1)	36 (24.3)	0.663
cancer (gastroenterogy)	8 (7.5)	13 (8.8)	0.819
cancer (without gastroenterogy)	3 (2.8)	11 (7.4)	0.163
Liver disease	9 (8.4)	0 (0.0)	<0.0001
inflammatory bowel disease	5 (4.7)	0 (0.0)	0.012
kidney disease	3 (2.8)	4 (2.7)	1.000
pulmonary disease	3 (2.8)	5 (3.4)	1.000
cardiovascular disease	4 (7.5)	13 (8.8)	0.132
diabetes	3 (2.8)	5 (3.4)	1.000
organic brain disease	3 (2.8)	0 (0.0)	0.073
cerebrovascular disease	3 (2.8)	5 (3.4)	1.000
dimentia	11 (10.3)	16 (10.8)	1.000
psycho‐neurologic disease	4 (7.5)	3 (2.0)	0.458
aging	25 (23.4)	30 (20.3)	0.644
others	10 (9.3)	11 (7.4)	0.647
not particular diseae	6 (5.6)	24 (16.2)	0.010
unknown	8 (7.5)	5 (3.4)	0.159

#### Type of ONS (review Q2)

**Fig 2:**
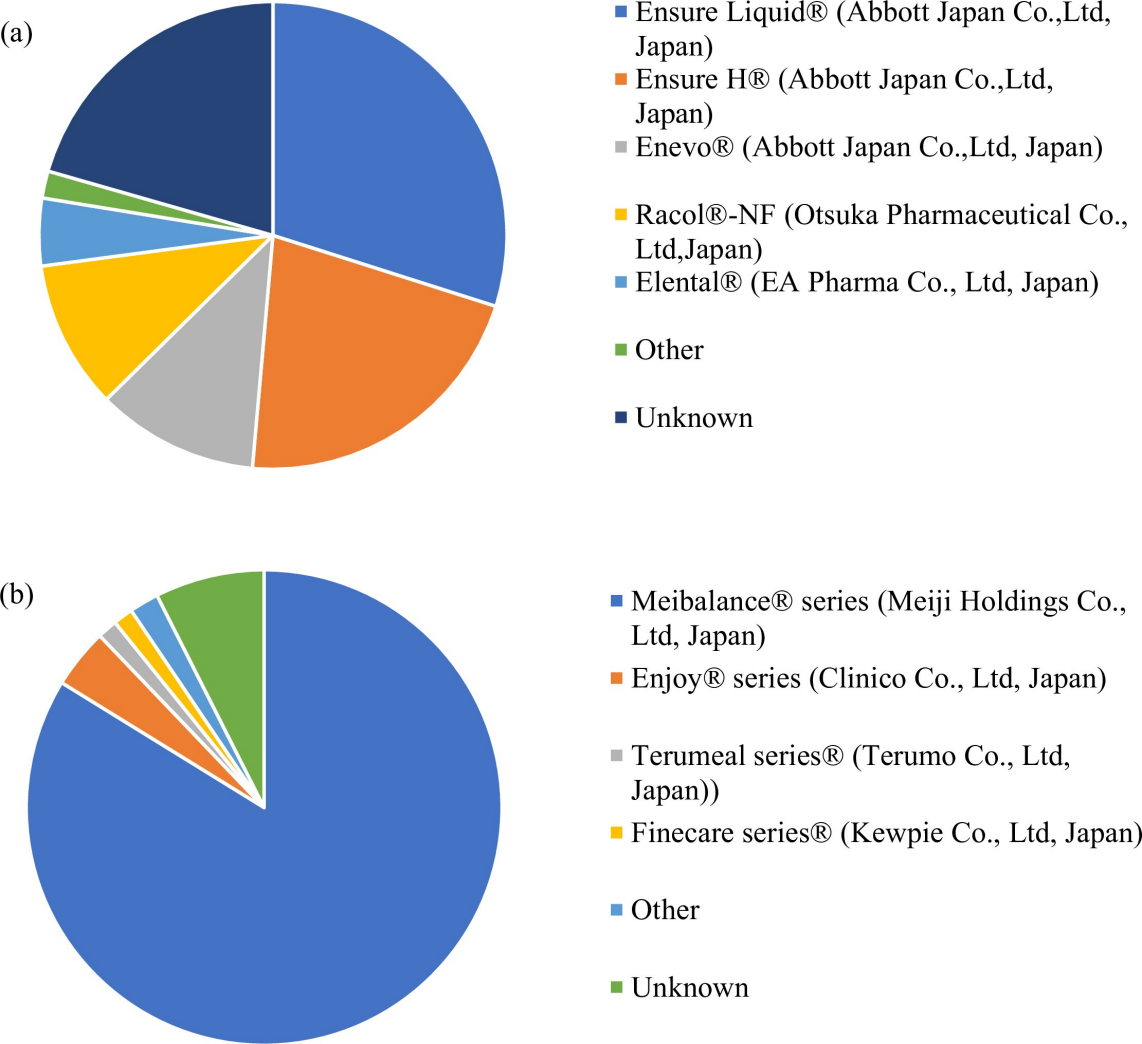
ONS types: (a) prescription ONS (n = 107). (b) over-the-counter ONS (n = 148).

Fig 2 shows the types of prescription and over-the-counter ONS. The types of prescription ONS were as follows: Ensure Liquid® (Abbott Japan Co., Ltd., Japan) in 32 patients (29.9%), Ensure H® (Abbott Japan Co., Ltd.) in 23 patients (21.5%), Enevo® (Abbott Japan Co., Ltd.) in 12 patients (11.2%), Racol®-NF (Otsuka Pharmaceutical Co., Ltd., Japan) in 11 patients (10.3%) and unknown in 22 patients (20.6%) (Fig 2A). The majority of the over-the-counter ONS (124 patients [83.8%]) were of the Meibalance^®^ series (Meiji Holdings Co., Ltd.,Japan), and other over-the-counter ONS were taken by under 5% of the over-the-counter ONS group (Fig 2B).

### Cost of ONS (review Q3)

The total monthly cost for prescription ONS was 3009±3486 JPY, and that for over-the-counter ONS was 3638±5124 JPY. No significant differences were noted between the groups (p = 0.127) (Table 6).

**Table 6:** The result of the survey questions (Q3,4).

	Prescription ONS (n = 107)	Over-the-counter ONS (n = 148)	p value
Cost (JPY per month) for ONS (Q3)			
cost for ONS (JPY/month), mean±SD	3009±3486	3638±5124	0.127
<1,000,n (%)	28 (26.2)	20 (13.5)	0.109
1,000–2,000,n (%)	21 (19.6)	30 (20.3)	
2,000–3,000,n (%)	13 (12.1)	30 (20.3)	
3,000–4,000,n (%)	13 (12.1)	22 (14.9)	
4,000–5,000,n (%)	3 (2.8)	4 (2.7)	
5,000–6,000,n (%)	17 (15.9)	22 (14.9)	
>6,000,n (%)	12 (11.2)	20 (13.5)	
Recommended to take ONS (Q4), n (%)			
prescribing doctor	72 (67.3)	-	-
other doctor	5 (4.7)	-	-
doctor	-	40 (27.0)	-
registered dietitian	2 (1.9)	21 (14.2)	0.001
pharmacist	10 (9.3)	10 (6.8)	0.485
Nurse	10 (9.3)	20 (13.5)	0.332
physical therapist / occupational therapist	3 (2.8)	4 (2.7)	1.000
helper/caremanager/care worker	11 (10.3)	24 (16.2)	0.200
others (medical personnel)	1 (0.9)	4 (2.7)	0.402
family	9 (8.4)	32 (21.6)	0.005
friend	6 (5.6)	9 (6.1)	1.000
nobody recommended	8 (7.5)	44 (29.7)	<0.0001
other	2 (1.9)	0 (0.0)	0.175

### Recommendation for ONS (review Q4)

In the prescription ONS group, 72 patients (67.3%) received a recommendation from their prescribing doctors, and 5 patients (4.7%) received a recommendation from other doctors. In the over-the-counter ONS group, 40 patients (27.0%) received a recommendation from a doctor. Recommendations were received from registered dietitians, helpers/care managers/care workers and family more frequently in the over-the-counter ONS group than in the prescription ONS group. Forty-four (29.7%) patients in the over-the-counter ONS group were not recommended from others and 8 (7.5) patients in the prescription ONS group. There were significant differences in the region between the two groups. (p<0.0001) (Table 6).

### Medical advice for ONS (review Q5-8)

In the prescription ONS group, all patients were given medical advice by doctors or registered dietitians, whereas in the prescription ONS group, 46 patients (31.1%) were given advice by doctors or registered dietitians (p<0.001). There were no marked differences between the groups in the advice about taking ONS, such as the number of times, amount or duration (Table 7).

**Table 7:** Medical advice for ONS.

	Prescription ONS (n = 107)	Over-the-counter ONS (n = 46)	p value
Number of times (Q6)			
Two or more times a day	18 (16.8)	8 (17.4)	0.663
Once a day	44 (41.4)	17 (37.0)	
Two to three times a week	10 (9.3)	4 (8.7)	
Once a week	1 (0.9)	3 (6.5)	
When you have a low food intake	27 (25.2)	11 (23.9)	
Not determined	4 (3.7)	2 (4.3)	
Unknown	3 (2.8)	1 (2.2)	
Amount (Q7)			
Indicated capacity	87 (81.3)	34 (73.9)	0.277
As much as possible	6 (5.6)	7 (15.2)	
Not determined	6 (5.6)	2 (4.3)	
Unknown	8 (7.5)	3 (6.5)	
Duration (Q8)			
≤2 weeks	11 (10.3)	5 (10.9)	0.218
>2 weeks to 1 month	8 (7.5)	4 (8.7)	
>1–3 months	18 (16.8)	7 (15.2)	
>3–6 months	10 (9.3)	3 (6.5)	
>6–12 months	6 (5.6)	0 (0.0)	
>12 months	12 (11.2)	4 (8.7)	
Not determined	34 (31.8)	23 (50.0)	
Unknown	8 (7.5)	0 (0.0)	

### Number of times, calories and amount of ONS (review Q9-11)

**Fig 3:**
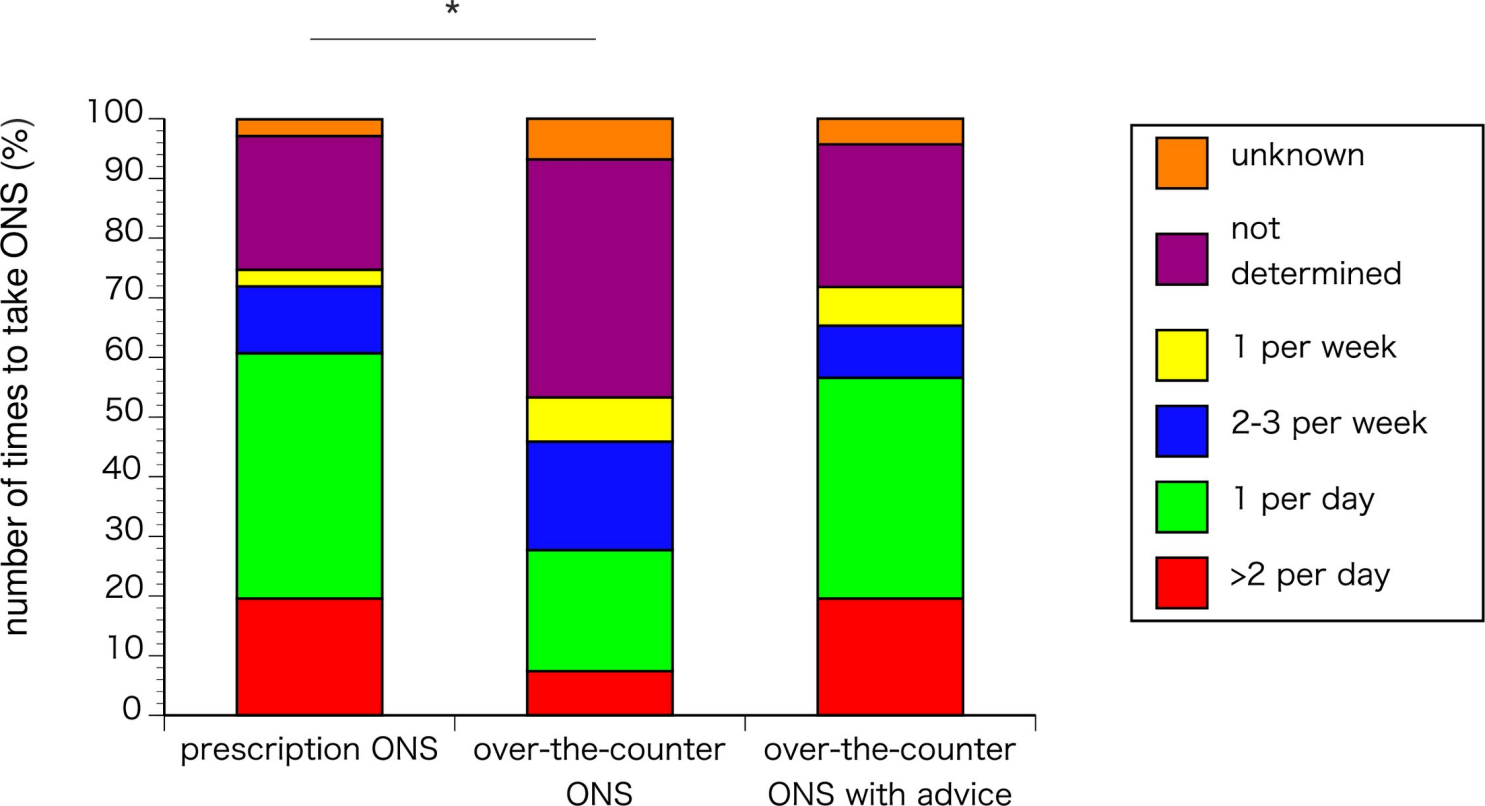
Number of times to take ONS. *p<0.0001.

The prescription ONS group consumed ONS significantly more often than the over-the-counter ONS group (p<0.0001). However, among those given advice by doctors or registered dietitians, there were no significant differences between the groups (p = 0.6253) (Fig 3).

Excluding patients who did not know the amount or type of ONS they consumed, the daily number of ONS calories in the prescription ONS group (n = 74) was significantly higher than in the over-the-counter ONS group (n = 122) (298.02±208.61 vs. 202.62±110.41, p = 0.00044) (Table 8).

**Table 8:** Energy intake.

	Prescription ONS (n = 74)	Over-the-counter ONS (n = 122)	p value
Energy intake (Q10) (kcal/day), mean±SD	298.0±208.6	202.6±110.4	<0.0001
<100, n (%)	13 (17.6)	20 (16.4)	<0.0001
101–200, n (%)	15 (20.3)	94 (77.0)	
201–300, n (%)	22 (29.7)	0 (0.0)	
301–400, n (%)	13 (17.6)	2 (1.6)	
>400, n (%)	11 (14.9)	6 (4.9)	

**Fig 4:**
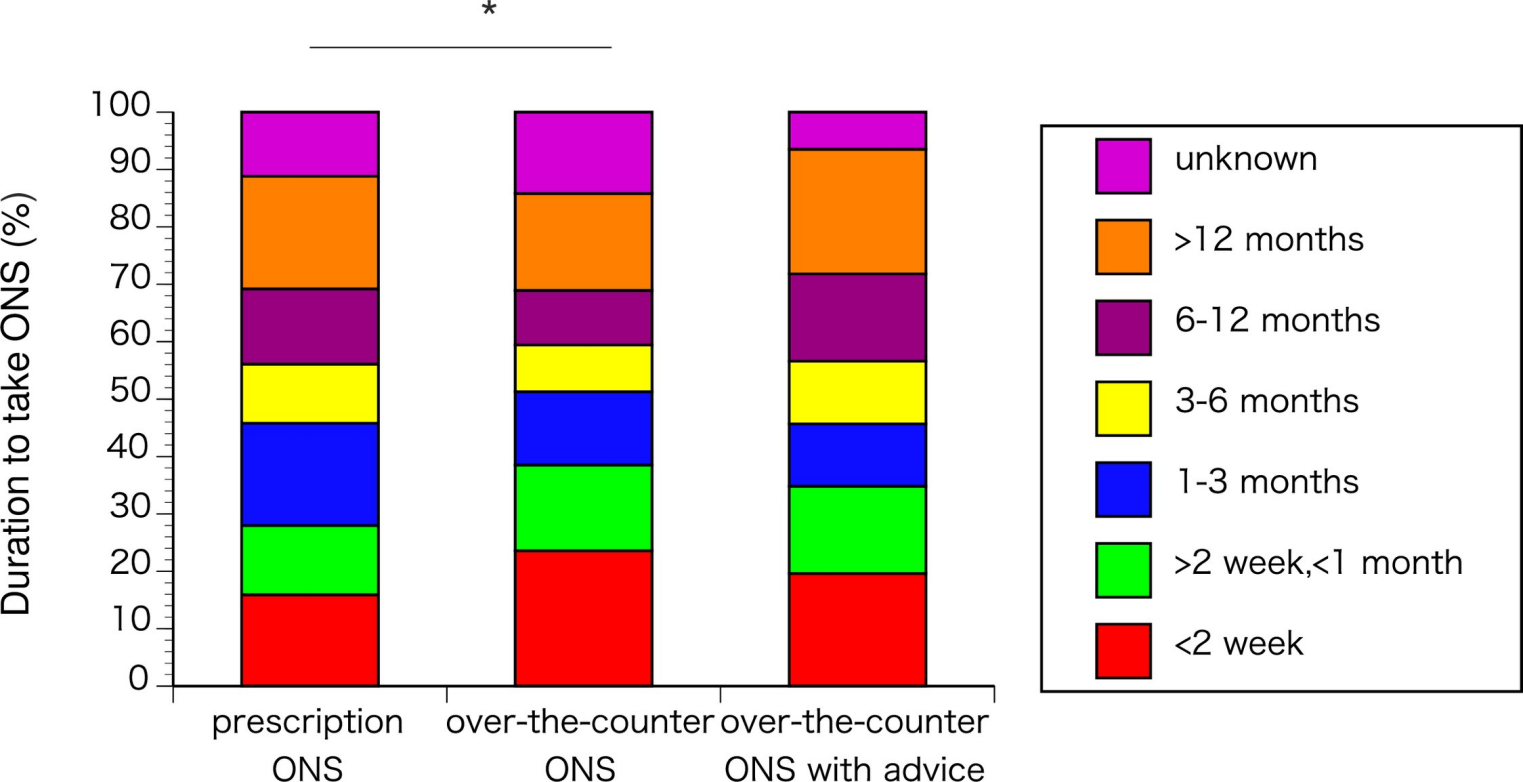
Duration taking ONS. *p<0.05.

In the prescription ONS group, the duration of taking ONS was significantly longer than in the over-the-counter ONS group (p<0.05). However, among those given advice by doctors or registered dietitians, there were no significant differences between the groups (p = 0.812) (Fig 4).

### Following medical advice and support for ONS (review Q12-14)

**Fig 5:**
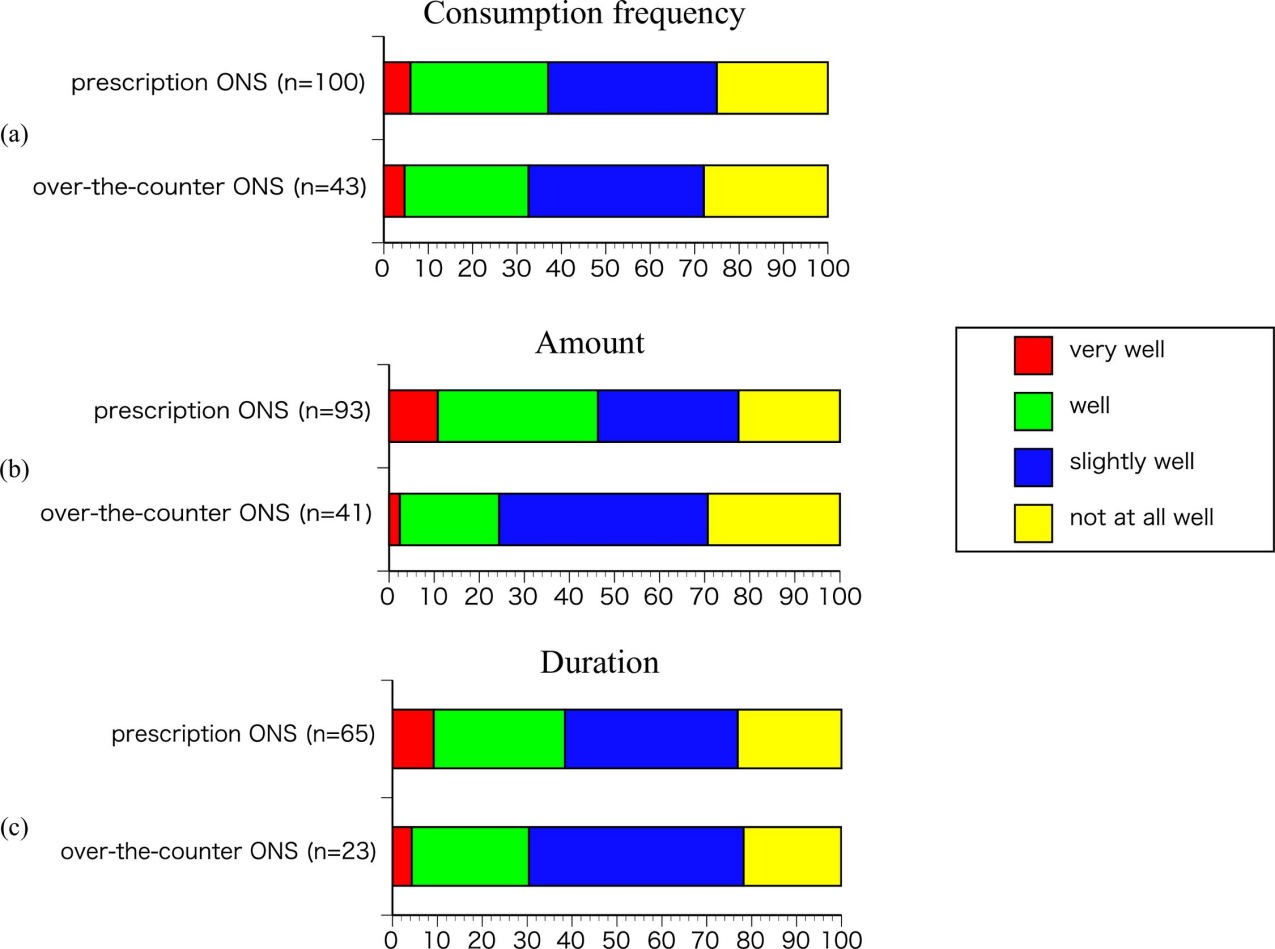
Adherence to medical advice on taking ONS. (a); Consumption frequency in the prescription ONS group (n = 100) and over-the-counter ONS group (n = 43). (b) Amount in the prescription ONS group (n = 93) and over-the-counter ONS group (n = 41). (c); Duration in the prescription ONS group (n = 65) and over-the-counter ONS group (n = 23).

Regarding adherence to medical advice on taking ONS, no significant differences were seen regarding adherence to consumption frequency in the prescription ONS group (n = 100) and over-the-counter ONS group (n = 43) (Fig 5A), amount in the prescription ONS group (n = 93) and over-the-counter ONS group (n = 41) (Fig 5B) and duration in the prescription ONS group (n = 65) and over-the-counter ONS group (n = 23) (Fig 5C).

Fifty patients in the prescription ONS group responded about why they did not adhere to the medical advice, as follows: doesn’t taste good, 12 patients (24.0%); too much to drink, 10 patients (20.0%); difficult to consume, 13 patients (26.0%); side effects, 6 patients (12.0%); don’t want to take, 7 patients (14.0%); forget to take, 12 patients (24.0%); not supported by family, 6 patients (12.0%); not supported by medical team, 1 patient (2.0%); and other reasons, 3 patients (6.0%).

Fifteen patients in the over-the-counter ONS group responded about why they did not adhere to the medical advice, as follows: doesn’t taste good, 5 patients (33.3%); too much to drink, 2 patients (13.3%); difficult to consume, 3 patients (20.0%); side effects, 2 patients (13.3%); don’t want to take, 4 patients (26.7%); forget to take, 4 patients (26.7%); not supported by family, 1 patient (6.7%); not supported by medical team, 2 patients (13.3%); and other reasons, 4 patients (26.7%).

The patients in the prescription ONS group who had provided support for continuing taking ONS were significantly more than those of the over-the-counter ONS group who had provided support (48 patients [44.9%] vs. 25 patients [16.9%] p<0.001).

### Satisfaction with ONS (review Q15)

**Fig 6:**
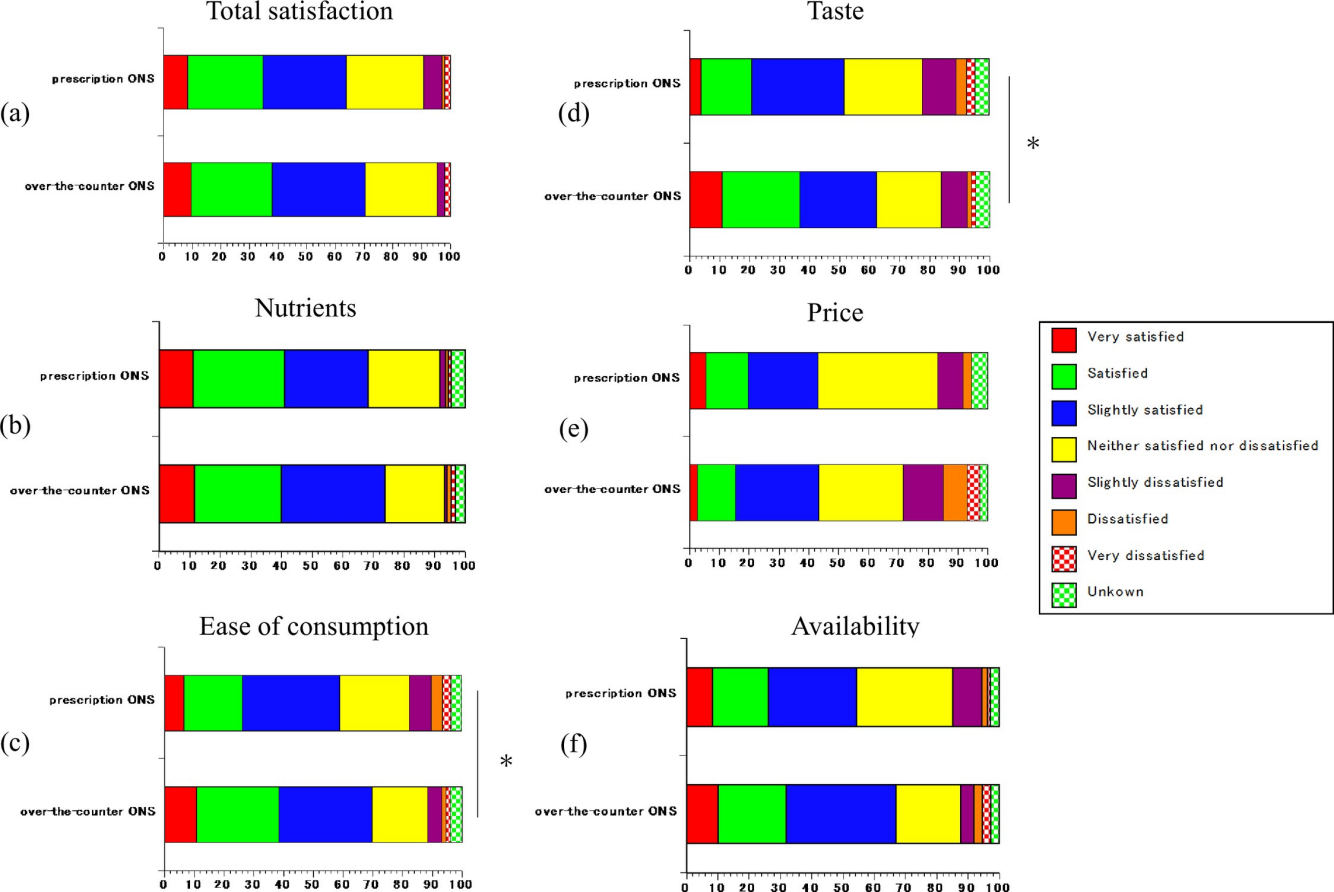
Satisfaction with ONS. (a); Overall satisfaction, (b); Nutrition, (c); Ease of consumption, (d); Taste, (e); Price, (f); Availability. *p<0.05.

Reports of satisfaction with ease of consumption (Fig 6C) and taste (Fig 6D) were significantly more frequent in the over-the-counter ONS group than in the prescription ONS group (p<0.05 and p<0.05, respectively). However, no significant differences were noted in the overall satisfaction, nutrition, price or availability (Fig 6).

### Awareness of other ONS (review Q16-18)

**Fig 7:**
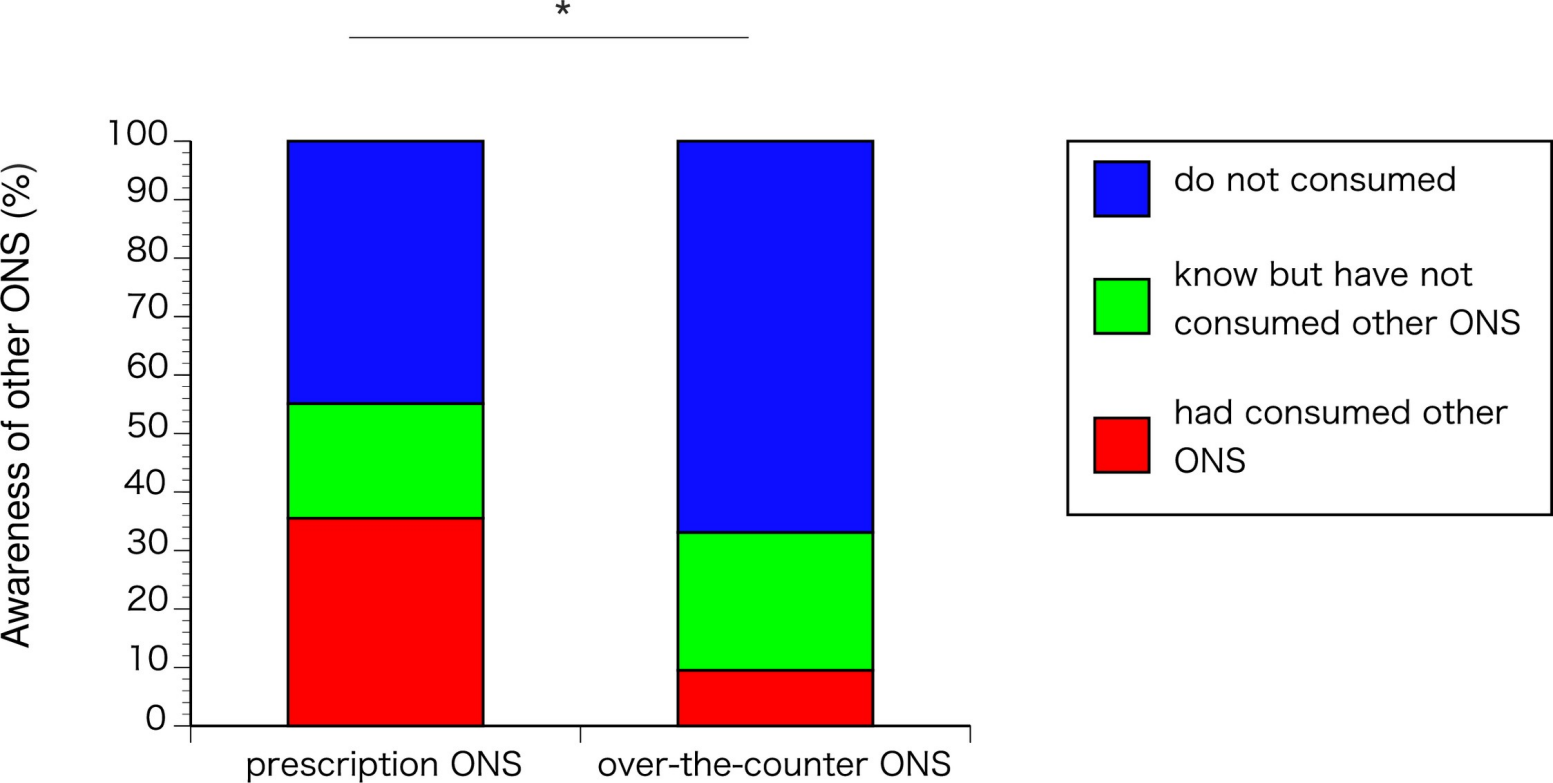
Awareness of other ONS. *p<0.0001.

The awareness of other ONS was significantly different between the groups (p<0.0001). In the prescription ONS group, 59 patients (55.1%) were aware of the existence of over-the-counter ONS, and 38 (35.5%) had consumed over-the-counter ONS. In contrast, in the over-the-counter ONS group, 49 patients (33.1%) knew about prescription ONS, and 14 (9.5%) had consumed prescription ONS. Fewer patients were unaware of the alternative in the over-the-counter ONS group than in the prescription ONS (48 patients [44.9%] vs. 99 patients [66.9%]; Fig 7).

**Fig 8:**
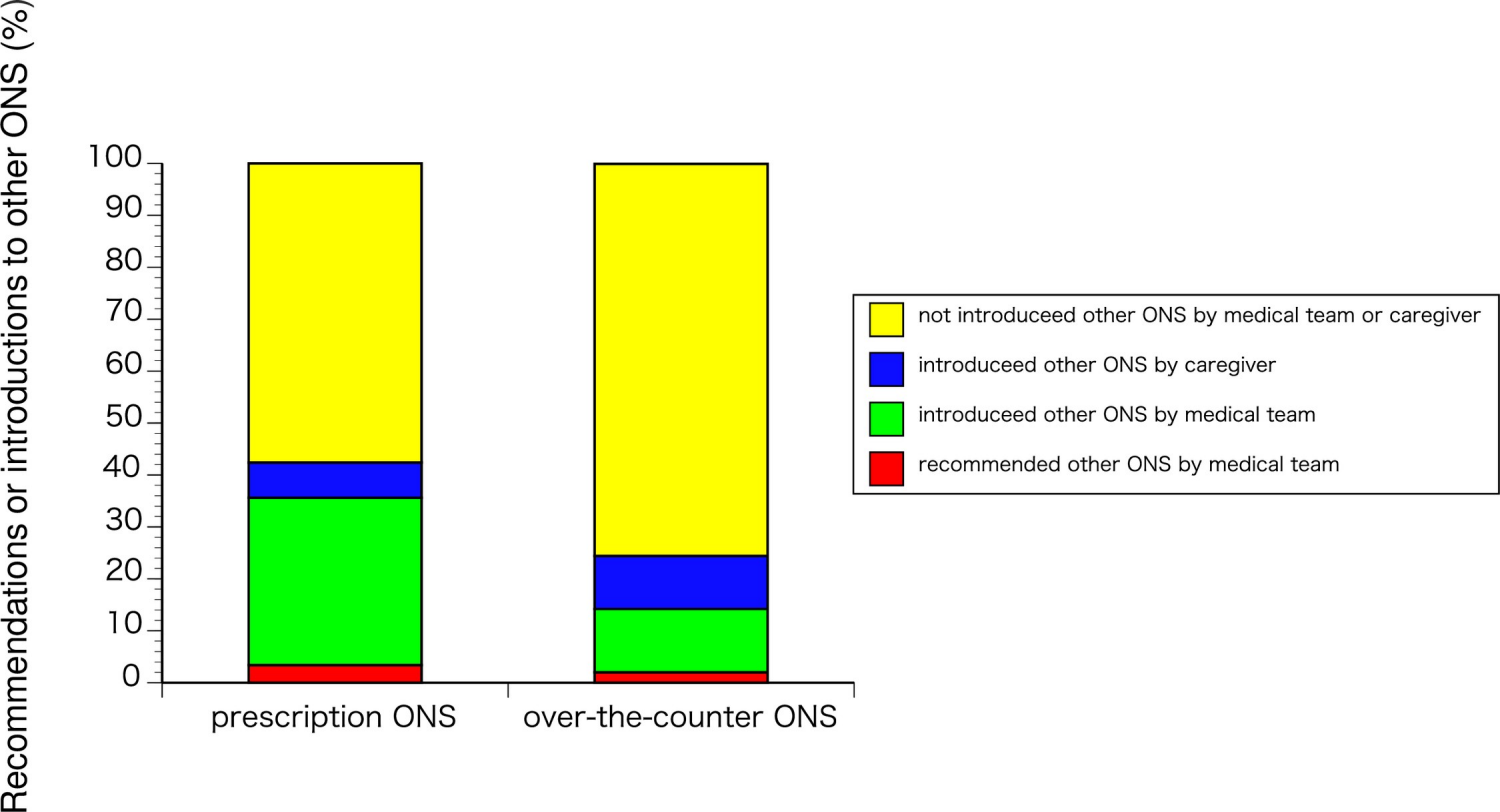
Recommendations or introductions to other ONS. Prescription ONS (n = 59) and Over-the-counter ONS (n = 49).

Among the patients who were aware of other ONS (prescription ONS group [n = 59], over-the-counter ONS group [n = 49]), there was no significant difference between the groups in the rate of being recommended or introduced to another ONS (p = 0.091). Thirty-four patients (57.6%) in the prescription ONS group had not been introduced to over-the-counter ONS by their medical team or caregiver, compared with 37 patients (75.5%) in the over-the-counter ONS group (Fig 8).

Among the patients in the prescription ONS group who knew about over-the-counter ONS, responses to “Why do you take your prescription ONS?” were as follows: recommended by a doctor, 14 patients (23.7%); recommended by medical team without doctors, 2 patients (3.4%); medical insurance coverage, 5 patients (8.5%); low price, 4 patients (6.8%); prescription drug, 4 patients (6.8%); easy to drink, 2 patients (3.4%); tasty, 1 patient (1.7%); no reason, 5 patients (8.5%). Among the patients in the over-the-counter ONS group who knew about prescription ONS, responses to “Why do you take your prescription ONS?” were as follows: advised by a doctor, 2 patients (4.1%); low price, 3 patients (6.8%); easy to drink, 5 patients (3.4%); tasty, 1 patient (1.7%); no reason, 5 patients (8.5%).

## Discussion

The prevalence of disease-related malnutrition is reportedly 20%–50% among patients admitted to hospitals [14] and 19% among hospital outpatients [23]. The condition is associated with a decreased quality of life [24,25] and increased length of hospital stay, morbidity, mortality [14,26] and cost of care [26,27].

Baldwin et al. conducted a systematic review and meta-analysis of nutritional intervention with dietary advice and/or oral nutritional supplements during treatment for cancer patients who were malnourished or at nutritional risk. Thirteen studies that included 1414 cancer patients were included in the analysis. Nutritional intervention resulted in statistically significant improvements in weight and energy intake, although no marked differences were observed after removing the studies responsible for heterogeneity. Some aspects of the quality of life, including emotional functioning, dyspnea, loss of appetite, and global quality of life, were improved. Nutritional intervention had no effect on mortality [28]. Concerning advancing age, undernutrition and chronic diseases, Gariballa et al. reported a randomized, double-blind, placebo-controlled trial of ONS. ONS of acutely ill patients improved their nutritional status and led to a statistically significant reduction in the number of non-elective readmissions [29].

For enhancing the food intake, dietary modification and food fortification are necessary. When these measures prove to be ineffective, the provision of ONS is indicated. Good adherence to ONS is essential to the success of nutritional therapy. Regarding factors influencing ONS adherence, a positive association has previously been shown between energy density and ONS adherence [21]. Other influencing factors are suggested to be the duration of ONS usage [30], variety of supplements prescription [21,30], how the supplement is taken [20] and whether or not the patient has been informed of the purpose of the ONS [14].

In the present study, in the prescription ONS group, all patients were given medical advice from doctors or registered dietitians, whereas in the prescription ONS group, only 46 patients (31.1%) were given such advice. Although all patients in the prescription ONS group received a prescription, 67.3% of patients were recommended ONS by their prescribing doctor, and 4.7% were recommended it by another doctor. This seems to suggest that some patients were recommended a prescription ONS by the nutrition support team and others by a multidisciplinary team. Intensive patient education by a nutrition support team to increase the number of feeding opportunities in order to cover the small amount tolerated per occasion as well as for patients to adhere to an adequate ONS program will help reduce body weight loss [31].

In order to achieve compliance, ONS comes in a variety of flavors and textures and can be served at different temperatures, according to patients' tastes, at times they prefer. Furthermore, energy-dense supplements seem to be more easily accepted and effective, as they minimize the volume that must be consumed in order to achieve the desired results [20,32]. Hubbard et al. reported in their systematic review of adherence to ONS that compliance across a heterogeneous group of unmatched studies was positively associated with a greater ONS energy density and total energy intakes, negatively associated with age and unrelated to the amount or duration of ONS prescription [20,32].

Reducing the volume of ONS during medication rounds increased the compliance of patients needing ONS [33]. In the present study, over 80% of over-the-counter ONS were Maibalance^®^. This formulation is based on the Dietary Reference Intakes for Japanese (2015), comes in several flavors, and can be ingested in small amounts with high calorie counts (200 kcal/125 ml). It is easy to purchase over the counter in supermarkets or pharmacies as well as online. Patients in the over-the-counter ONS group reported significantly higher satisfaction with the ONS ease of consumption and taste than those in the prescription ONS group. However, the number of ONS calories consumed in a day was significantly higher in the prescription ONS group than in the over-the-counter ONS group. It is not possible to consume a sufficient amount of the over-the-counter ONS by taking in patients’ own determined, which might be related to the significant difference in the BMI between the two groups.

There has been increasing interest in the effects of ONS on healthcare use and costs. In the acute setting, reductions in the length of hospital stay and complications and a reduction in associated costs have been well documented with ONS [1]. Furthermore, regarding the total healthcare cost and quality of life among patients, ONS use significantly reduces the rate of hospital readmission, especially in older adults [34]. In the present study, while there was no significant difference between the two groups, the cost in the over-the-counter ONS group was higher than in the prescription ONS group. In Japan, through the national health insurance scheme, patients under 70 years old only have to pay a 30% medical copayment, while those 70–74 years old must pay 20%, and those ≥75 years old must pay 10% (those with income comparable to the current workforce have a copayment of 30%) [35]. If the adherence and calories intake of the two groups were similar, over-the-counter ONS would be much higher than prescription ONS.

In this study, there was a significant difference in the regional spread between the two groups. This may be because doctors and registered dietitians in some regions might recommend one ONS over another, or patients’ purchase intentions in certain regions might be higher than in others.

The present study is associated with some limitations due to its wide age distribution and varied health conditions among respondents and small sample size. As this study used an online cross-sectional survey, it was limited to subjects who had internet access. Patients freely consumed both the ONS and ordinary food. Although the data on the ONS intake were accumulated with the utmost care, data on the actual dietary caloric intake were not collected. This study did not categorize ONS into subtypes, such as oligomeric, disease-specific and macronutrient ONS. It might therefore have included patients who did not require ONS treatment, especially the over-the-counter ONS group. It seems also crucial to ensure that patients are offered flavors, textures and/or scents that they like, as these can influence their compliance [36]. Our study did not assess the protein, mineral or vitamin content. In a systematic review and meta-analysis, high-protein supplements were shown to produce clinical benefits, with subsequent economic implications [23]. Furthermore, not only calories but also protein content can significantly influence results.

## Conclusion

Adherence can be improved by encouraging patients and explaining the reasoning and aims of nutritional support. Overall, a greater support by the medical team is still needed in order to maximize adherence to supplementation, especially concerning the calories, timing and period, so that benefits can be achieved and sustained. Consequently, more studies are needed in order to understand the effects of ONSs.

## References

[1] Stratton RJ, Elia M. Encouraging appropriate, evidence-based use of oral nutritional supplements. Proc Nutr Soc. 2010;69(4):477–87. Epub 2010/08/10. 10.1017/S0029665110001977 . 20696091

[2] Gomes F, Schuetz P, Bounoure L, Austin P, Ballesteros-Pomar M, Cederholm T, et al ESPEN guidelines on nutritional support for polymorbid internal medicine patients. Clin Nutr. 2018;37(1):336–53. Epub 2017/07/24. 10.1016/j.clnu.2017.06.025 . 28802519

[3] Koretz RL, Avenell A, Lipman TO, Braunschweig CL, Milne AC. Does enteral nutrition affect clinical outcome? A systematic review of the randomized trials. Am J Gastroenterol. 2007;102(2):412–29; quiz 68. 10.1111/j.1572-0241.2006.01024.x . 17311654

[4] Ferreira IM, Brooks D, White J, Goldstein R. Nutritional supplementation for stable chronic obstructive pulmonary disease. Cochrane Database Syst Rev. 2012;12:CD000998 Epub 2012/12/12. 10.1002/14651858.CD000998.pub3 . 23235577 PMC11742366

[5] National Institute for Health and Clinical Excellence (NICE). Nutrition Support for Adults: Oral Nutrition Support, Enteral Tube Feeding and Parenteral Nutrition (Clinical guideline 32). London, UnitedKingdom:National Collaborating Centre for Acute Care; 2006. 21309138

[6] Stratton RJ, Green CJ, Elia M. Disease-Related Malnutrition: An Evidence-BasedApproach to Treatment.Cambridge,MA: Cabi Publishing; 2003.

[7] Milne AC, Potter J, Vivanti A, Avenell A. Protein and energy supplementation in elderly people at risk from malnutrition. Cochrane Database Syst Rev. 2009;(2):CD003288 Epub 2009/04/15. 10.1002/14651858.CD003288.pub3 . 19370584 PMC7144819

[8] Baldwin C, Weekes CE. Dietary advice with or without oral nutritional supplements for disease-related malnutrition in adults. Cochrane Database Syst Rev. 2011;(9):CD002008 Epub 2011/09/07. 10.1002/14651858.CD002008.pub4 21901680 PMC6465043

[9] Chima CS, Barco K, Dewitt ML, Maeda M, Teran JC, Mullen KD. Relationship of nutritional status to length of stay, hospital costs, and discharge status of patients hospitalized in the medicine service. J Am Diet Assoc. 1997;97(9):975–8; quiz 9–80. 10.1016/S0002-8223(97)00235-6 . 9284874

[10] Somanchi M, Tao X, Mullin GE. The facilitated early enteral and dietary management effectiveness trial in hospitalized patients with malnutrition. JPEN J Parenter Enteral Nutr. 2011;35(2):209–16. 10.1177/0148607110392234 . 21378250

[11] Lawson RM, Doshi MK, Barton JR, Cobden I. The effect of unselected post-operative nutritional supplementation on nutritional status and clinical outcome of orthopaedic patients. Clin Nutr. 2003;22(1):39–46. . 12553948 10.1054/clnu.2002.0588

[12] Beattie AH, Prach AT, Baxter JP, Pennington CR. A randomised controlled trial evaluating the use of enteral nutritional supplements postoperatively in malnourished surgical patients. Gut. 2000;46(6):813–8. 10.1136/gut.46.6.813 10807893 PMC1756438

[13] Gariballa S, Forster S. Effects of dietary supplements on depressive symptoms in older patients: a randomised double-blind placebo-controlled trial. Clin Nutr. 2007;26(5):545–551. 10.1016/j.clnu.2007.06.007 17662509

[14] Gariballa S, Forster S, Walters S, Powers H. A randomized, double-blind, placebo-controlled trial of nutritional supplementation during acute illness. Am J Med. 2006;119(8):693–9. 10.1016/j.amjmed.2005.12.006 . 16887416

[15] Norman K, Pichard C, Lochs H, Pirlich M. Prognostic impact of disease-related malnutrition. Clin Nutr. 2008;27(1):5–15. Epub 2007/12/03. 10.1016/j.clnu.2007.10.007 . 18061312

[16] Jensen MB, Hessov I. Dietary supplementation at home improves the regain of lean body mass after surgery. Nutrition. 1997;13(5):422–30. 10.1016/s0899-9007(97)91280-6 . 9225334

[17] Gosney M. Are we wasting our money on food supplements in elder care wards? J Adv Nurs. 2003;43(3):275–80. 10.1046/j.1365-2648.2003.02710.x . 12859786

[18] McMurdo ME, Price RJ, Shields M, Potter J, Stott DJ. Should oral nutritional supplementation be given to undernourished older people upon hospital discharge? A controlled trial. J Am Geriatr Soc. 2009;57(12):2239–45. Epub 2009/11/17. 10.1111/j.1532-5415.2009.02568.x . 19925613

[19] Gammack JK, Sanford AM. Caloric supplements for the elderly. Curr Opin Clin Nutr Metab Care. 2015;18(1):32–6. 10.1097/MCO.0000000000000125 . 25474011

[20] Hubbard GP, Elia M, Holdoway A, Stratton RJ. A systematic review of compliance to oral nutritional supplements. Clin Nutr. 2012;31(3):293–312. Epub 2012/01/17. 10.1016/j.clnu.2011.11.020 . 22257636

[21] Bauer J, Capra S, Battistutta D, Davidson W, Ash S, Group CCS. Compliance with nutrition prescription improves outcomes in patients with unresectable pancreatic cancer. Clin Nutr. 2005;24(6):998–1004. Epub 2005/09/06. 10.1016/j.clnu.2005.07.002 . 16140426

[22] Ministry of Health, Labour and Welfare. 2nd NDB Open Data Japan 2017 http://www.mhlw.go.jp/stf/seisakunitsuite/bunya/0000177221.html

[23] Cawood AL, Elia M, Stratton RJ. Systematic review and meta-analysis of the effects of high protein oral nutritional supplements. Ageing Res Rev. 2012;11(2):278–96. Epub 2011/12/22. 10.1016/j.arr.2011.12.008 . 22212388

[24] Lis CG, Gupta D, Lammersfeld CA, Markman M, Vashi PG. Role of nutritional status in predicting quality of life outcomes in cancer—a systematic review of the epidemiological literature. Nutr J. 2012;11:27 Epub 2012/04/24. 10.1186/1475-2891-11-27 22531478 PMC3408376

[25] Norman K, Kirchner H, Lochs H, Pirlich M. Malnutrition affects quality of life in gastroenterology patients. World J Gastroenterol. 2006;12(21):3380–5. 10.3748/wjg.v12.i21.3385 16733855 PMC4087869

[26] Correia MI, Waitzberg DL. The impact of malnutrition on morbidity, mortality, length of hospital stay and costs evaluated through a multivariate model analysis. Clin Nutr. 2003;22(3):235–9. . 12765661 10.1016/s0261-5614(02)00215-7

[27] Freijer K, Tan SS, Koopmanschap MA, Meijers JM, Halfens RJ, Nuijten MJ. The economic costs of disease related malnutrition. Clin Nutr. 2013;32(1):136–41. Epub 2012/07/10. 10.1016/j.clnu.2012.06.009 . 22789931

[28] Baldwin C, Spiro A, Ahern R, Emery PW. Oral nutritional interventions in malnourished patients with cancer: a systematic review and meta-analysis. J Natl Cancer Inst. 2012;104(5):371–85. Epub 2012/02/15. 10.1093/jnci/djr556 . 22345712

[29] Gariballa S, Forster S, Walters S, Powers H. A randomized, double-blind, placebo-controlled trial of nutritional supplementation during acute illness. Am J Med. 2006;119(8):693–9. 10.1016/j.amjmed.2005.12.006 . 16887416

[30] Nieuwenhuizen WF, Weenen H, Rigby P, Hetherington MM. Older adults and patients in need of nutritional support: review of current treatment options and factors influencing nutritional intake. Clin Nutr. 2010;29(2):160–9. Epub 2009/10/13. 10.1016/j.clnu.2009.09.003 . 19828215

[31] Lee HO, Han SR, Choi SI, Lee JJ, Kim SH, Ahn HS, et al Effects of intensive nutrition education on nutritional status and quality of life among postgastrectomy patients. Ann Surg Treat Res. 2016;90(2):79–88. Epub 2015/01/28. 10.4174/astr.2016.90.2.79 26878015 PMC4751149

[32] Hubbard GP, Elia M, Holdoway A, Stratton RJ. A systematic review of compliance to oral nutritional supplements. Clin Nutr. 2012;31(3):293–312. Epub 2012/01/17. 10.1016/j.clnu.2011.11.020 . 22257636

[33] van den Berg GH, Lindeboom R, van der Zwet WC. The effects of the administration of oral nutritional supplementation with medication rounds on the achievement of nutritional goals: a randomized controlled trial. Clin Nutr. 2015;34(1):15–9. Epub 2014/04/30. 10.1016/j.clnu.2014.04.011 . 24880907

[34] Stratton RJ, Hebuterne X, Elia M. A systematic review and meta-analysis of the impact of oral nutritional supplements on hospital readmissions. Ageing Res Rev. 2013;12(4):884–97. Epub 2013/07/24. 10.1016/j.arr.2013.07.002 . 23891685

[35] Ministry of Health, Labour and Welfare. Health and Medical Services. Health Insurance http://www.mhlw.go.jp/english/policy/health-medical/health-insurance/index.html (accessed on 15 July 2019)

[36] Paccagnella A, Morassutti I, Rosti G. Nutritional intervention for improving treatment tolerance in cancer patients. Curr Opin Oncol. 2011;23(4):322–30. 10.1097/CCO.0b013e3283479c66 . 21552123

