# Supplementary material for: Adherences to oral nutritional supplementation among hospital outpatients: An online cross-sectional survey in Japan
Source: PLoS One. 2019 Sep 26;14(9):e0222972. doi: 10.1371/journal.pone.0222972 (PMC6762170; doi:10.1371/journal.pone.0222972)
Supplement: Attachment — Submitted filename: Dear reviewer.docx [file pone.0222972.s001.docx]

Dear reviewers

Thank you for your some comments.We wish to express our appreciation to the reviewers for their insightful comments on our paper. The comments have helped us significantly improve the paper.

Dear reviewer #1

Q1; Clearly state the objective of the study in the introduction section.

Add importance of oral nutritional supplements in the introduction section.

A1; We added the section in introduction.

“The aims of this study were the examination of difference between outpatients used prescription ONS and those used over-the-counter ONS and adherence to prescription ONS prescribed by a doctor and to over-the-counter ONS purchased by themselves.”

“A variety of benefits have been found for ONS use, including reduced length of stay, inpatient episode cost, complication rates, depressive symptoms, readmission rates, and improved lean body mass recovery.”

Q2; Table 3 is too long. If possible, divide into two or more parts.

A2; Table3 was divided two parts (Table 3 and Table 4)

Q3; Table 4 is too long. If possible, divide into two or more parts.

A3; Table4 was divided two parts (Table 5 and Table 6)

Q4; More statistical parameters are applied for the authentication of data.

A4; The statistical analysis for 2 groups was used statistically general method according to the question format.

Q5; The references are arranged according to the guidelines of journal.

A5; Absolutely, we check the guidelines of journal.

Reviewer #2:

Q1; The study design section has some results which can be move to the results section.

A1; We moved to the result about the percentage of patients in NDB and survey duration.

Reviewer #3:

Q1; The manuscript should be revised for linguistic errors.

A1; This paper was revised by licensed English native speaker.

Q2; The oral nutritional supplementation should be specified within the manuscript.

A2; We added the sentence in method.

“Prescription ONS were registered in Japan as follows; Elental^®^ (EA Pharma Co., Ltd , Japan), Elental P^®^ (EA Pharma Co., Ltd , Japan), Ensure Liquid^®^ (Abbott Japan Co., Ltd., Japan), Ensure H^®^ (Abbott Japan Co., Ltd.), Enevo^®^ (Abbott Japan Co., Ltd.), Twinline-NF^®^ (Otsuka Pharmaceutical Co., Ltd., Japan), Racol-NF^®^ (Otsuka Pharmaceutical Co., Ltd., Japan). Over-the-counter ONS are registered as foodstuffs ONS in Japan.”

Q3; The survey duration is too short and patients number is small.

A3; The number of samples that could be sufficiently quantitatively analyzed was 300 or more in this study. The duration was the period required to have the answer.

Reviewer #4: SI have the following questions/suggestions, which I hope the authors will consider:

Q1; From what I could understand, what the authors call the “prescribed” ONS is also purchased by the patients. Similarly, what they call the “purchased” ONS may also be prescribed/recommended by nutritional experts. I found the terminology confusing. Since the difference between the two categories is that one is available with prescription only while the other is available over-the-counter, I recommend calling the “prescribed” and “purchased” ONS as “prescription” and “over-the-counter” ONS, respectively.

A1; I edited prescribed to prescription, and purchased to over-the-counter

Q2; On the first page of the abstract, second to the last line, it starts as, “In contrast, in the prescribed ONS group, only 46 patients …”. Did the authors mean purchased ONS group instead?

A2; Absolutely, I edited.

Q3; The large majority of individuals who started the questionnaire did not complete it. Is there any systematic reason why this was the case?

A3; Firstly, we collect the patients who visited a hospital for some illness within the past year and currently consume ONS as a hospital outpatient. For collecting 300 patients, we need to start the questionnaire to the large majority of individuals.

Q4. All the figure legends are repetitions of text also in the main text. I suggest that the figure legends be replaced with a brief title.

A4; I edited to a brief title.

Reviewer #5:

Q1; How did you select participants to invitation to survey?

A1; I edited about the description of selected participants

“The survey was hosted by the market research company EPOCA Marketing Co., Ltd., which recruited samples from 2.2 million people registered with the company intended to be representative of the Japan population.”

Q2; Did you have any incentive to complete the survey

A2; The aims of this study were the examination of difference between outpatients used prescription ONS and those used over-the-counter ONS and adherence to prescription ONS prescribed by a doctor and to over-the-counter ONS purchased by themselves.

Q3; The main limitation of this study is the low response rate to survey. Therefore, the result cannot represent the nationwide data in Japan

A3; We excluded “nationwide survey”
